# Anomeric *O*-Functionalization of Carbohydrates for Chemical Conjugation to Vaccine Constructs

**DOI:** 10.3390/molecules23071742

**Published:** 2018-07-17

**Authors:** Simon S. Park, Hsiao-Wu Hsieh, Jacquelyn Gervay-Hague

**Affiliations:** Department of Chemistry, University of California, Davis, CA 95616, USA; sipark@ucdavis.edu (S.S.P.); hwhsieh@ucdavis.edu (H.-W.H.)

**Keywords:** carbohydrates, glycosylation, glycosides, glycoconjugates, anomeric functionalization

## Abstract

Carbohydrates mediate a wide range of biological interactions, and understanding these processes benefits the development of new therapeutics. Isolating sufficient quantities of glycoconjugates from biological samples remains a significant challenge. With advances in chemical and enzymatic carbohydrate synthesis, the availability of complex carbohydrates is increasing and developing methods for stereoselective conjugation these polar head groups to proteins and lipids is critically important for pharmaceutical applications. The aim of this review is to provide an overview of commonly employed strategies for installing a functionalized linker at the anomeric position as well as examples of further transformations that have successfully led to glycoconjugation to vaccine constructs for biological evaluation as carbohydrate-based therapeutics.

## 1. Introduction

### 1.1. Emergence of Glycomics

Nucleic acids, proteins and carbohydrates are three important classes of biomolecules. DNA, RNA and the proteins they encode have relatively confined connectivity and predictable chemistries owing to the limited number of ways the nucleic acid and amino acid building blocks can be combined. In contrast, carbohydrates have multiple similarly reactive hydroxyl groups that connect with varying region- and stereochemistries giving rise to a complex set of structures that have no corresponding genetic blueprint [1,2]. With the launch of the Human Genomic Project in the beginning of this century [3,4], efforts have shifted toward understanding structure/function relationships of post-translational modifications of proteins. Glycosylation is a prominent form of post-translational modification occurring in a majority of eukaryotic proteins [5,6,7]. Other biomolecules such as glycolipids and glycosylphosphatidylinositols (GPI anchor) display hydrophilic carbohydrate moieties that participate in ligand-receptor binding, cell-to-cell interactions and pathogenic processes such as bacterial and viral infection as well as cancer metastasis [8,9]. Deciphering the “sugar codes” created by specific sequences of oligosaccharides linked to lipid and protein anchors is an emerging area of glycomics, which like proteomics has the underlying goal of connecting chemical structures to biological functions. 

Understanding how structure gives rise to function is critical for the development of carbohydrate-based therapeutics and expedient access to synthetic materials is a significant challenge for researchers in this area. Like most natural products, glycoconjugates are typically difficult to isolate and may only occur as heterogenic mixtures in scarce amounts. Common isolation techniques often require enzymatic digestion, detergent extraction and multiple purifications, which may degrade the sugars; although recent methods to extract *O*-glycans using bleach hold promise for commercial use [10]. To address these limitations, synthetic platforms that afford large-scale production of pure and chemically defined glycoconjugates are under development. Chemoenzymatic methods offer a complementary approach; however, enzyme availability, substrate specificity and scalability can hinder product diversity. In either case, achieving the desired stereochemical specificity and multiplicity of ligation products with high purity remains a challenge [11,12,13]. One approach to conjugating carbohydrates to binding partners of interest (i.e., protein or lipid) in enantiomerically pure form is through the stereoselective installment of linkers with functionalizable handles, which can be elaborated to generate complex glycans and/or multivalent displays. 

### 1.2. Overview of Glycosylation Principles

**Figure 1:**
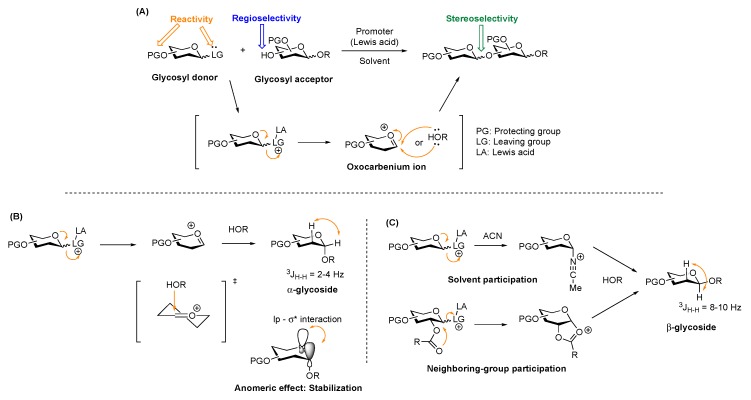
(**A**) Glycosylation reaction at a glance. (**B**) Mechanistic overview of α-glycosides. (**C**) Mechanistic overview of β-glycosides.

Carbohydrate synthesis requires a crafty approach in selecting protecting groups, participating groups, promoter systems, glycosyl donor and selectively deprotected glycosyl acceptors in order to achieve stereo-controlled glycosylation in reasonable yields [14,15] (Figure 1A). The reactivities of the carbohydrate and aglycon partners can be finely-tuned by introducing different protecting groups. According to the “arm-disarm” concept introduced by Fraser-Reid, electron releasing ether-type (i.e., silyl and benzyl) protecting groups arm the donor while inductively withdrawing ester (i.e., acetyl and benzoyl) disarm the donor [16,17]. Donor leaving groups can be activated using various promoters, which are commonly Lewis acids added in stoichiometric or catalytic amounts. Upon departure of the anomeric leaving group, the resulting oxocarbenium ion is ready for coupling with a glycosyl acceptor (nucleophile) to form the corresponding glycosidic bond. When the acceptor is another carbohydrate, oligosaccharide synthesis results. If the acceptor is a non-carbohydrate aglycon, a glycoconjugate is formed. The stereoselectivity at the anomeric position can be influenced by steric hindrance, the anomeric effect, and internal or external group participation (such as neighbor-group participation or solvent effect) [18,19] (Figure 1B,C). NMR is an essential tool used to determine the anomeric stereoselectivity. According to the Karplus equation, α/β-glycosides are characterized by the chemical shift of the anomeric proton, as well as the ^3^J_H,H_ and ^1^J_C,H_ coupling constants.[20] Concordantly, the ratio of products is measured by integration of the anomeric proton peaks. 

A wide-range of glycosyl donors are available in the synthetic chemist’s tool box including halides, imidates and thioglycosides. Each of these has advantages and drawbacks depending upon the synthetic strategies employed. Nevertheless, the underlying goal in all these endeavors is to achieve efficient assembly of glycoconjugates with high stereochemical integrity and to this end programmable one-pot glycosylations [21,22] and pre-activation one-pot glycosylations [23,24], together with automated solid-phase synthesis [25,26] have emerged as powerful platforms. Despite considerable efforts toward achieving efficient glycosylations, currently there is no general methodology for synthesizing oligosaccharides and glycoconjugates. 

### 1.3. Elements of Carbohydrate-Based Vaccine Research

**Figure 2:**
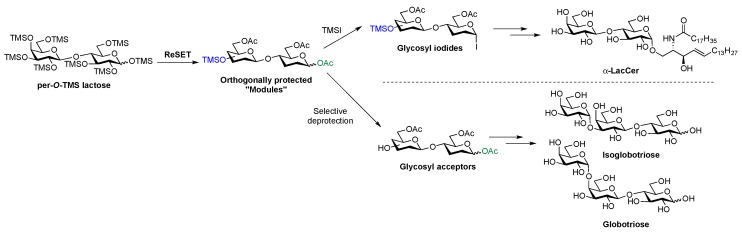
Regioselective Silyl Exchange Technology (ReSET) to streamline the total synthesis of glycolipids and tumor-associated carbohydrate antigens (TACAs).

Strategies to reduce the number of protecting group manipulations and to use common intermediates offer alternative ways of streamlining oligosaccharide synthesis [27]. For example, integrating regioselective silyl exchange technology (ReSET) [28,29] with glycosyl iodide glycosylation has afforded step-economical total syntheses of α-lactosylceramide (α-LacCer) and globo series tumor-associated carbohydrate antigens (TACAs) [30] (Figure 2). The methodology utilizes per-*O*-TMS-lactose to generate a library of partially acetylated/silylated building blocks in just two steps from free lactose. The resulting orthogonally protected “modules” can be transformed into either a glycosyl iodide or a selectively deprotected glycosyl acceptor for oligosaccharide assembly. Compared to previously published methods, which require 16–20 steps [31,32], the ReSET platform uses one-third of the synthetic steps, providing a solution for rapid, stereoselective synthesis of immunogenic glycolipids and TACAs. 

Tumor-associated carbohydrate antigens (TACAs) are a class of carbohydrate biomarkers expressed on tumor cells. The polar head group may be attached to a protein backbone and classified as a glycoprotein or a lipid anchor, such as ceramide, to constitute a glycolipid [33,34,35]. In either case, the stereochemistry at the glycosylation site is well defined because specific presentation geometry is required for biological recognition. Another salient feature of TACAs is that they are poorly immunogenic and typically are found in clusters on the cell surface, which increases the effective concentration of the carbohydrate recognition element. 

**Figure 3:**
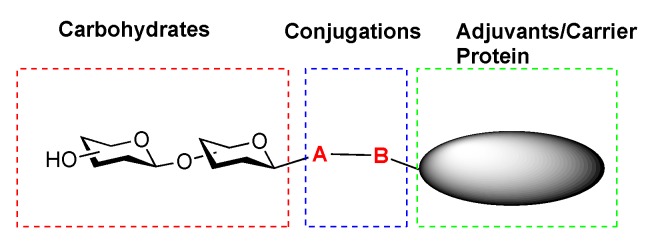
Elements of Carbohydrate-Based Vaccine Research.

Carbohydrate vaccine development encompasses three major elements of research: (1) efficient synthesis of complex carbohydrate recognition elements; (2) identification of protein/adjuvant carriers that elicit immune response; and (3) chemically compatible and stereoselective methodologies for conjugating carbohydrate antigens to the immunogenic protein (Figure 3). Aspects of each of these elements have been reviewed in the recent literature [26,31,36,37] The focus of this review is to highlight chemical methods for achieving stereoselective functionalization of the anomeric center with functionalized linkers that can be further elaborated for conjugation to vaccines carriers [38,39,40,41,42].

## 2. Stereoselective Anomeric Functionalization for Chemical Conjugation

**Figure 4:**
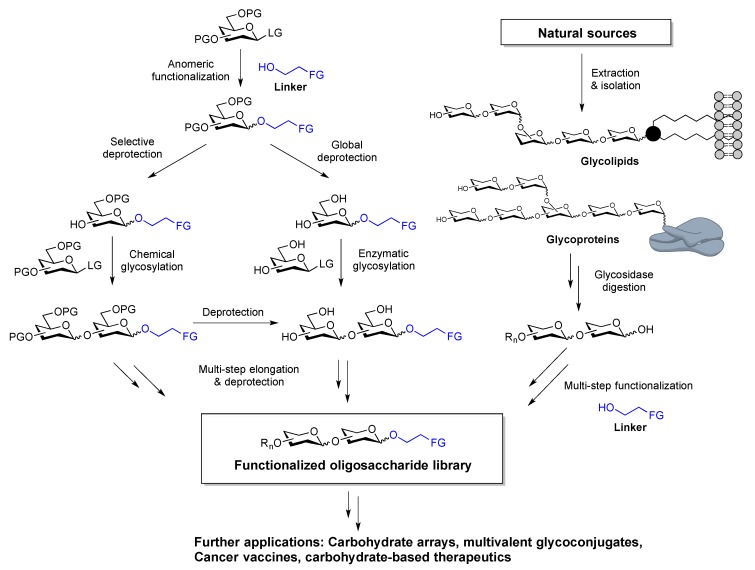
Anomeric functionalization strategies. PG: Protecting group; LG: Leaving group; FG: Functional group.

Introducing a functionalizable linker at the anomeric position is a common way to modify oligosaccharides for chemical conjugation. Often, the linker is introduced at the beginning of the synthesis, usually at the mono- or disaccharide stage (Figure 4). However, the functional group on the linker must tolerate all chemical transformations en route to the target compound. The starting oligosaccharide is either derived from chemical synthesis or isolated from natural sources by digestion of glycoproteins or glycolipids. In either case, chemical modification of a free oligosaccharide to ready it for conjugation requires multiple synthetic transformations and may be challenging due to the chemical vulnerability of interglycosidic linkages. The following sections provide an overview of two commonly employed linker functionalities, alkene and azide, and compatible glycosylation conditions. These two reactive groups were developed in 1990s and have gained in popularity because of their biological orthogonality and widespread application. 

### 2.1. Anomeric Functionalization with Terminal Alkenyl Linkers

**Figure 5:**
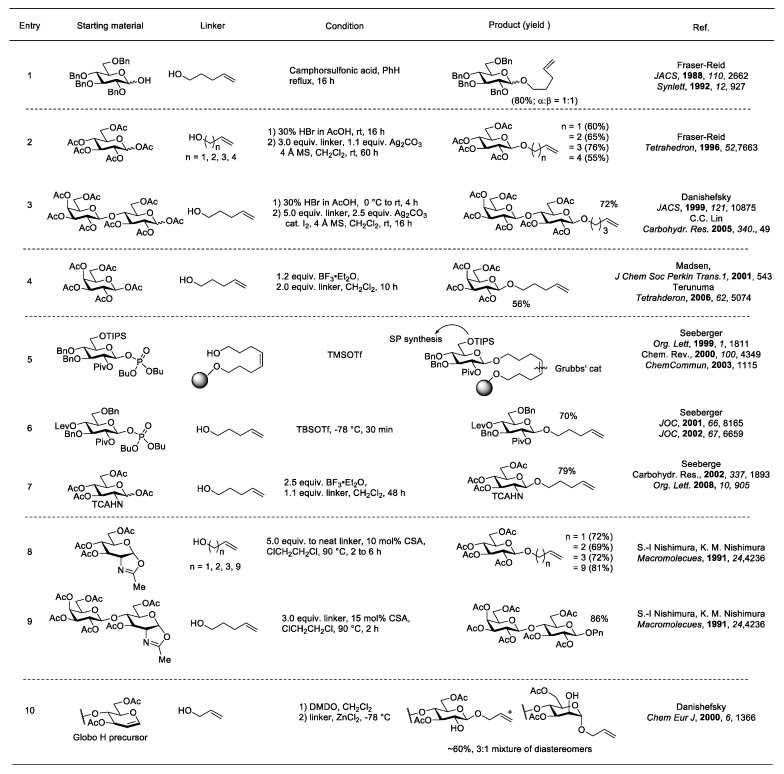
Terminal alkene linker glycosylations.

A literature survey of terminal alkene linkers incorporated into oligosaccharides is shown in Figure 5. The *n*-4-pentenyl linker was first introduced by the Fraser-Reid group in 1988 [43,44]. It was added to a benzylated glucose with an anomeric hemiacetal under acidic reflux conditions (Figure 5, entry 1). The Koenigs-Knorr type method led to a 1:1 α/β mixture of anomers in 80% yield, and the glycosylated product was used to explore the “arm-disarm” concept. The glycosylation of per-*O*-benzylated glucose afforded a higher yield compared to ester protected sugars (80% vs. high 60–70%), but the lack of neighboring group participation at C-2 led to a mixture of α/β anomers. Later on, the same group screened different terminal alkenyl linkers ranging from three to six carbons for coupling with per-*O*-acetylated glucosyl bromide with AgCO_3_ promotion [45] (Figure 5, entry 2). The results showed the *n*-4-pentenyl analog gave better yields among the alcohols of differing lengths. Bromide formation followed by AgCO_3_-promoted *n*-4-pentenol addition also occurred with per-*O*-acetylated lactose to obtain the corresponding β-lactoside in 72% yield [46,47] (Figure 5, entry 3). Silver-promoted glycosylations gave exclusive β-glycosides due to the neighboring group participation of the C-2 acetate. Optimal conditions usually required low temperatures to suppress side reactions, such as hydrolysis and acyl migration, and to increase the stereoselectivity. 

In an effort to develop metal free methodologies, Lewis acid-promoted conditions were then explored. Transformation of per-*O*-acetylated sugars to *n*-4-pentenyl sugars was achieved by the direct activation of an anomeric acetate using stoichiometric amounts of BF_3_·Et_2_O [48,49] (Figure 5, entry 4). Although this strategy alleviated the bromide formation step, yields were typically lower than the bromide method, presumably due to batch-to-batch variations of a key reagent, BF_3_·Et_2_O, which is typically found in 46–49% in commercial form and therefore requires fresh distillation prior to the reaction. 

In 1999, the Seeberger group developed a version of the 4-pentenyl glycosylation for solid phase oligosaccharide synthesis [50,51,52] (Figure 5, entry 5). An octenediol linker attached to solid support was coupled with a glycosyl phosphate using catalytic TMSOTf for activation. After the carbohydrate elongation process, the linker was released using olefin metathesis with Grubbs’ catalyst and the corresponding *n*-4-pentenyl glycoside was isolated via HPLC. The *n*-4-pentenyl linker not only functionalized the anomeric position, but also served as a leaving group under the activation of proper promoters [53]. The dual property of the linker was found very useful in traditional total synthesis of oligosaccharides published by the same group [54,55] (Figure 5, entry 6). Instead of using acetate at the C-2 position for neighboring group participation, the Seeberger group used the pivaloyl (Piv) group for this purpose, since the Piv group is less likely to form orthoester side products. Similarly, the trichloroacetate (TCA) protecting group was also used to protect nitrogen-containing sugars, such as glucosamine, to prevent oxazoline formation [56,57] (Figure 5, entry 7). 

Oxazoline formation is a common byproduct of *N*-Acetyl containing sugars due to the neighboring group participation. The relatively stable intermediates can be isolated without decomposition and can be used as glycosyl donors under proper conditions. In 1991, the Nishimura group demonstrated that oxazolines of glucose and lactose could be glycosylated with *n*-4-pentenol under heated, acidic conditions [58] (Figure 5, entries 8 and 9). However, harsh conditions leading to the cleavage of glycosidic linkages and yielding undesired products prevented this methodology from gaining popularity. 

The Danishefsky group is well known for developing glycals as carbohydrate donors in complex oligosaccharide syntheses [59]. Epoxidation of glucal with dimethyldioxirane (DMDO) give the α-1,2-epoxide, which undergoes glycosylation with 2-propenol in the presence of zinc catalyst. The glycosylation yields mostly β-linked alkenyl glycan in 60% yield.

#### Diversification of the Alkene Linker Functionality for Carbohydrate Conjugation to Carriers

**Figure 6:**
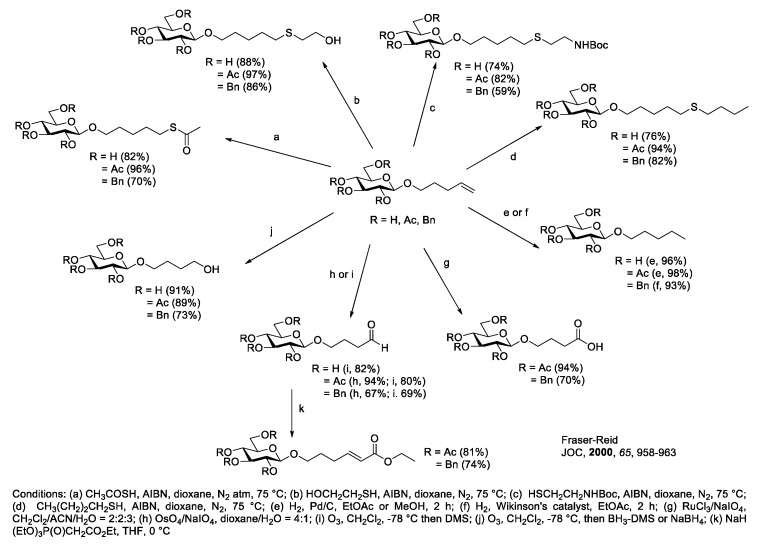
Functional diversification of terminal alkenyl linkers [60].

Further manipulation of terminal alkenyl linkers can be achieved through various conditions [60] (Figure 6). The double bond functionality can be transformed to the corresponding thioether via radical reaction using thiol derivatives. Hydrogenation using Pd and Wikinson’s catalyst affords saturated alkyl linkers. Oxidative cleavage using NaIO_4_ with RuCl_3_ and OsO_4_ yield the corresponding carboxylic acid and aldehyde which are readily available for ester bond formation, Wittig-type reaction, and reductive amination. Ozonolysis followed by hydroboration-oxidation can also be applied to alkenyl linker, leading to the corresponding alcohol.

Ozonolysis and reductive amination were employed in the preparation of Globo H conjugates. Globo H was isolated in 1984 by the Hakomori group [61]. It is composed of hexasaccharide β-linked to ceramide. The tumor-associated carbohydrate antigen (TACA) can be found overexpressed in breast, pancreas, small bowel, ovarian and prostate cancer [62]; therefore, it has been a valuable synthetic target toward novel therapeutic development. Isolating globo H from biological samples is challenging and typically limited to sub-milligram quantities. On the other hand, chemical synthesis has enabled large-scale production of homogenous and pure oligosaccharides in larger scale. 

**Figure 7:**
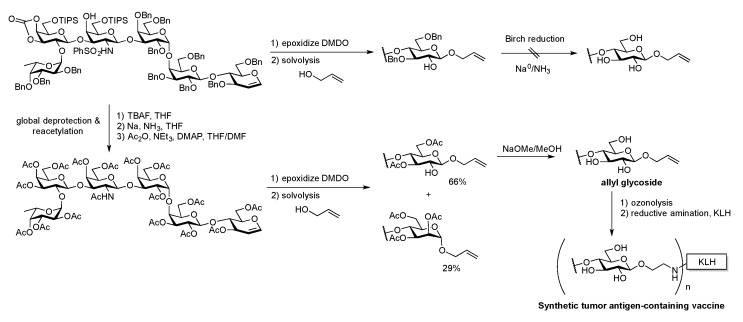
Danishefsky’s late stage introduction of an allyl linker and subsequent reductive amination with KLH carrier to prepare Globo H containing cancer vaccine candidate.

The Danishefsky group has led efforts toward fully synthetic carbohydrate-based anticancer vaccine development [63,64]. In the first generation of globo H total synthesis, the hexasaccharide was assembled via glycal chemistry, leaving a glycal functionality at the reducing end (Figure 7). The protected globo H glycal was subsequently reacted with dimethyldioxirane (DMDO) followed by solvolysis with allylic alcohol. The epoxide ring opening glycosylation reaction proceeded in good yield with b-selective incorporation of the alkenyl linker. However, this functionality did not survive the subsequent deprotection step using the Birch reduction. The crude reaction mixture contained mainly glycosidic bond cleavage products and fragmentation of the hexasaccharide [65,66,67].

To avoid these complications, the globo H glycal was deprotected and then reprotected as a per-*O*-acetylated trisaccharide prior to the DMDO reaction. Epoxide ring opening glycosylation with allylic alcohol solvolysis under the activation of ZnCl_2_ led to a 66% yield of the desired β-glycoside, but a significant amount of the α-glycoside was also found (29% yield). Saponification of the major per-acetylated β-*O*-allyl globo H glycoside afforded a fully deprotected globo H trisaccharide functionalized with an alkenyl linker in quantitative yield. Ozonolysis of the alkene followed by reductive amination with the KLH carrier protein, gave a fully synthetic carbohydrate vaccine for the immunology investigations (Figure 7). 

**Figure 8:**
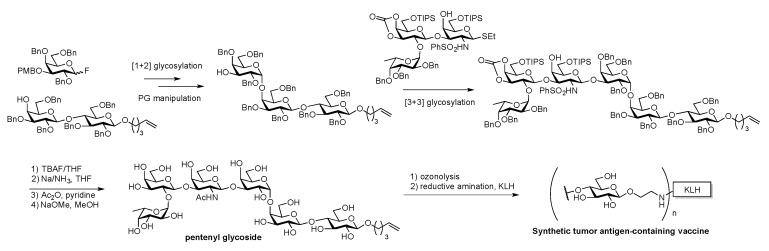
Danishefsky’s 2nd generation total synthesis of Globo H cancer vaccine: Early stage introduction of 4-pentenyl linker.

The group also developed a second-generation total synthesis of globo H by introducing a 4-pentenyl linker at an early stage of the lactosyl building block preparation (Figure 8). The linker was not only orthogonal to both [1+2] and [3+3] glycosylation conditions but also stable enough to undergo both TBAF desilylation and Birch reduction. The fully deprotected 4-pentenyl glycoside could also be subjected to the same ozonolysis/reductive amination conditions to conjugate KLH carrier protein [66]. 

**Figure 9:**
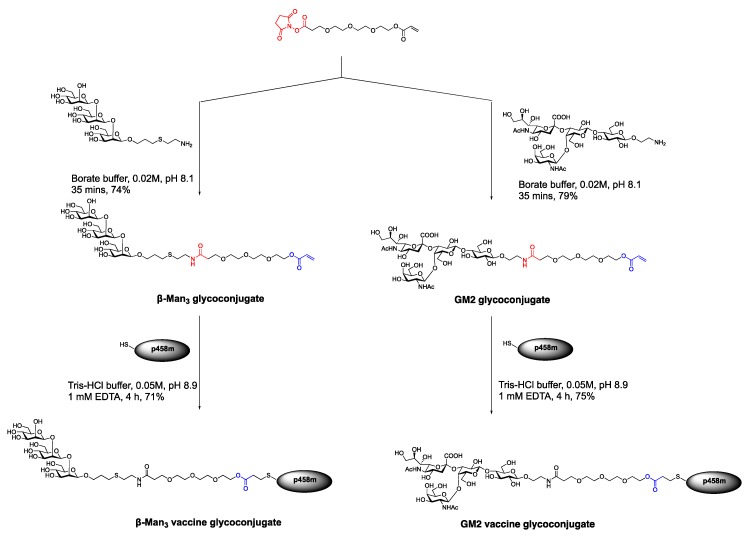
Synthesis of conjugate vaccine candidates composed of Man_3_ or GM2 and T_H_ cell peptide epitope (p458m) by the Bundle group [68].

In 2008, the Bundle group developed a non-immunologenic triethylene oxide linker equipped with an NHS ester on one end and an alkenyl functional group on the other [68]. This linker was then attached to amine functionalized oligosaccharides including β-1,2 mannan and ganglioside series GM2 upon treatment with an aqueous borate buffer (0.02M) at pH 8.1 in 74–79% yields (Figure 9). 

The glycoconjugate linker retained an alkenyl handle that could be further reacted with a sulfhydryl group of a peptide construct containing the specific T_H_ peptide epitope (p458m) in high yields. Remarkably, the β-Man_3_ glycoconjugates can be synthesized post-glycosylation in an aqueous buffer and be recognized by Man_3_ specific antibody [68]. 

**Figure 10:**
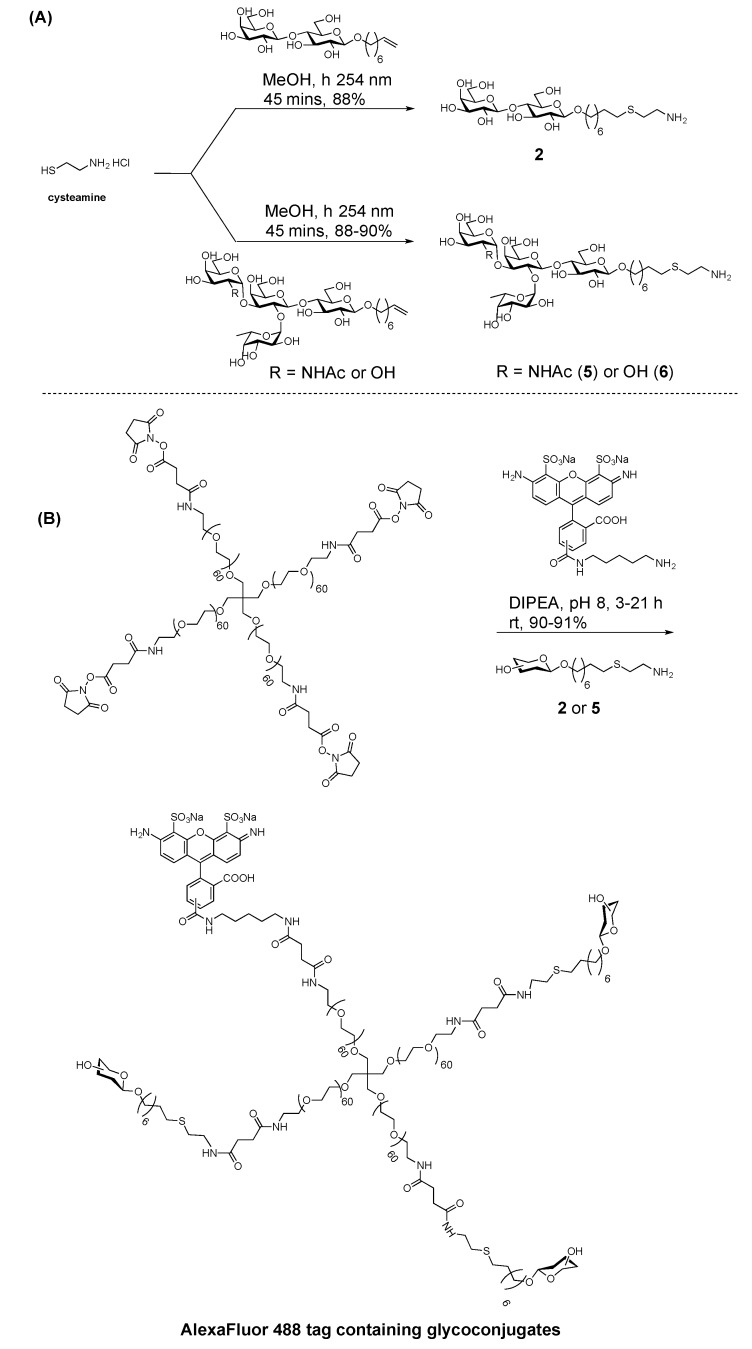
Synthesis of multivalent ABO blood group glycoconjugates on a PEG scaffold developed by the Cairo group. (**A**) Synthesis of amine-terminated glycans (**B**) Synthesis of a heterotrifunctional AlexaFluor 488 containing trivalent glycotope using iterative amine conjugation [69].

More recently, the Cairo group employed thio-alkenylation conjugation chemistry to construct multivalent ABO blood group glycoconjugates [69] on a PEGylated scaffold. The design for generating tetravalent or trivalent forms of ABO blood glycoconjugates was based on various conjugation chemical strategies. It first started with an octenyl lactoside, which was converted to an amine under photo-induced radical addition of cysteamine hydrochloride. The resulting amine-terminated glycan was then conjugated to an NHS PEG scaffold. In order to construct fluorescently labeled glycoconjugates, the authors used iterative amine chemistry to attach the AlexaFluor 488 tag followed by the addition of amine-terminated glycans in 90–91% yields (Figure 10). 

**Figure 11:**
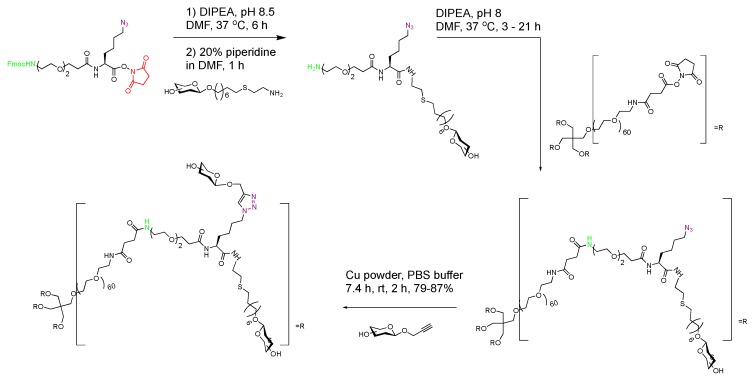
Synthesis of multivalent ABO blood group glycoconjugates using heterotrifunctional PEG linker developed by the Cairo group [69].

In addition, the authors crafted a heterotrifunctional linker equipped with NHFmoc, azido, and NHS ester groups (Figure 11). The first group of the amine-terminated glycan was reacted with the NHS ester of the linker followed by the removal of NHFmoc to expose an amine, which was reacted with the PEG scaffold. With the azide functionality intact, a second sialoglycan was attached by CuAAc (vide infra) to generate octavalent heterobifunctional glycoconjugates in >78% yields with molecular weights ranging from 15.5–18.1 kDa (Figure 11). An extension of the conjugation chemistry with deprotected glycans explored by the Bundle group was applied to a heterotrifunctional linker to generate a large, complex glycoconjugate in a controlled manner in good yields. This work was a more straightforward approach than the earlier work by the Buriak group, which demonstrated the feasibility of conjugating *p*-nitrophenyl functionalized glycan antigens on silica microparticles albeit in lower molecular size [70]. With careful manipulation of the bio-orthogonal functional groups available on a robust, non-immunologenic linker, large glycotopes could be synthesized in pure forms.

In general, terminal alkene linkers, especially the *n*-4-pentenyl linker, have been popular in the carbohydrate chemistry community since the late 1980 to 2000s. While thiolinker was first introduced to study glycosylation reactions, it later proved to be a versatile functional group for total synthesis of complex glycans equipped with a functionalizable linker. One drawback of the linker is the electron-rich nature of the double bond functionality. It does not survive strongly acidic conditions, Lewis acid-abundant environments, radical reactions or hydrogenation, which limits the use of certain reagents during oligosaccharide syntheses. Partly due to the limitations of terminal alkenyl linkers in conjugation applications, the concept of bioorthogonal chemistry has been advanced by the Bertozzi laboratory to explore the next generation of reactions that could be performed under physiological conditions without losing reactivity and selectivity [71]. For these purposes, azido linkers have been explored and continue to garner interest in the area of chemical glycobiology. 

### 2.2. O-Anomeric Functionalization with Terminal Azide Linkers

Click chemistry was first described by Sharpless and co-workers in 2001 as the term for biocompatible, highly reactive and atom economical reactions [72]. Among the reactions that fulfill the criteria of click chemistry and bioorgonal chemistry, the copper-catalyzed azide-alkyne cycloaddition (CuAAC) (i.e., ‘click’) has been widely applied in the synthesis of biologically important molecules. In fact, this reaction has become synonymous to click chemistry. Many modifications of the azido-alkyne cycloaddition reaction have been investigated including the introduction of terminal azido linkers at the sugar anomeric position [73]. 

**Figure 12:**
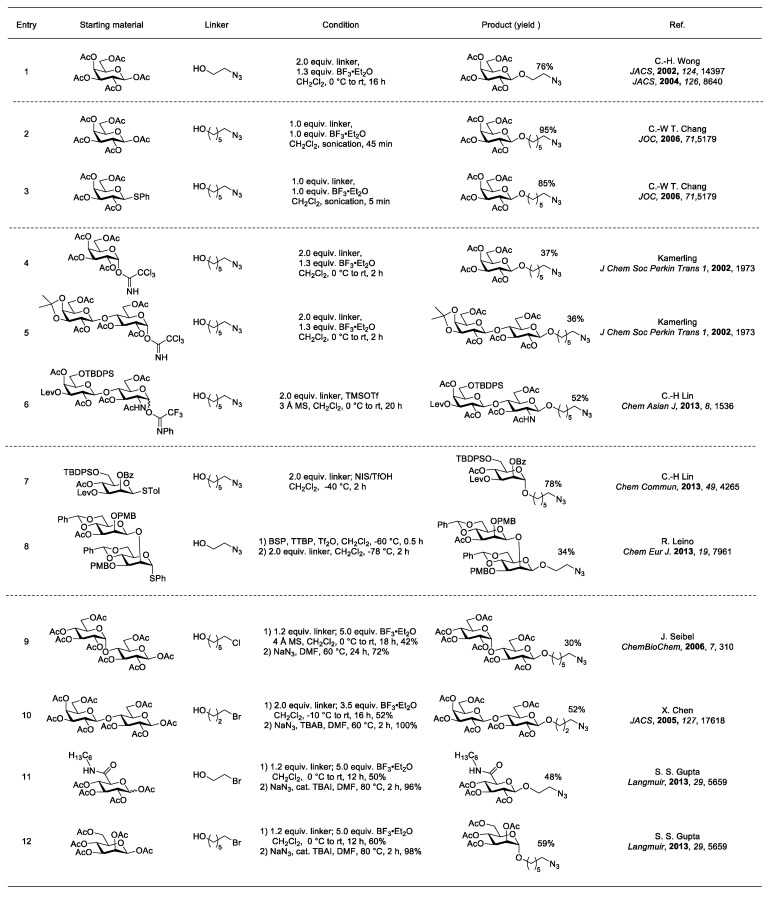
Terminal azide linker glycosylations.

An azido alcohol linker was utilized by the Wong laboratory for sugar anomeric functionalization [74,75] (Figure 12, entry 1). In the reaction, 2-azidoethanol was coupled with per-*O*-acetylated galactose using stoichiometric BF_3_·Et_2_O to form β-2-azidoethyl galactoside in 76% yield. Besides the 2-azidoethyl linker, a 6-azidohexyl linker was also used in anomeric functionalization. The higher C to N ratio made the 6-azidohexyl linker a safer choice compared to its two carbon counterpart [76,77]. 

Interestingly, the length of the azido linker affected the glycosylation results—the longer the linker, the less reactive it was under glycosylation conditions. To hasten the process, BF_3_·OEt_2_-promoted glycosylations were sonicated using either the anomeric acetate or thiophenyl donors [78] (Figure 12, entries 2 and 3), which helped reduce the reaction time from 16 h to less than 60 min with increased yield to 85–95%. 

Trichloroacetimidates are reliable donors for glycosylation and can easily be prepared from acetates in two steps, which involve deacetylation and basic imidate formation [14]. Glycosylation under the activation of BF_3_·OEt_2_ to form β-6-azidohexyl glycoside was not very successful, leading to only around a 30% yield [79] (Figure 12, entries 4 and 5). The low glycosylation yields have been attributed to the disarmed nature of the per-*O*-acetylated donors and the nucleophilicity of the leaving group, which can lead to *N*-glycosylated side products [80]. *N*-Phenyltrifluoroacetimidate was invented to avoid these issues and has been applied to a variety of natural glycoside syntheses [81]. The glycosylation result of *N*-phenyltrifluoroacetimidate with 6-azidohexyl linker under the activation of catalytic TMSOTf was demonstrated to have higher yields than its tricholoracetimidate analogues and could be extended to aminodisaccharides, such as protected *N*-acetyllactosamine [82] (Figure 12, entry 6). 

Thioglycosides, such as thiophenyl and thiotolyl glycosides, are stable and easy to handle with long shelf-life. They have proven useful in challenging glycosidic bond forming reactions, including mannosyl glycosides. In 2013, Lin’s group demonstrated thiotolyl mannoside could be coupled with a 6-azidohexyl linker under the activation of NIS/TfOH at low temperature to afford α-6-azidohexyl manoside in good yield [83] (Figure 12, entry 7). On the other hand, a more challenging β-azidoethyl mannoside was synthesized by Leino’s group using Crich’s modified pre-activation [84] method, which required very careful temperature control [85] (Figure 12, entry 8). Although the yield of the desired product was only 34%, the applicability of this methodology to disaccharide thioglycoside donors is notable.

**Figure 13:**
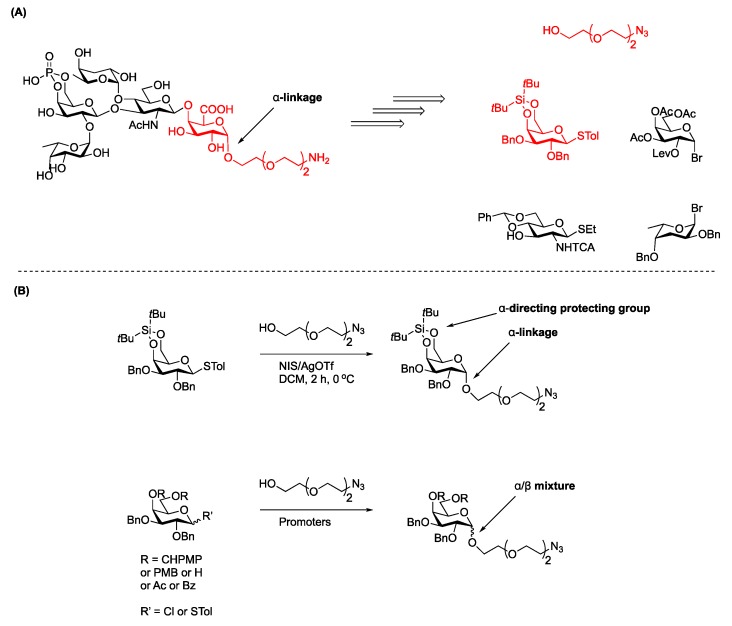
Kovac synthesis of oligosaccharide fragments of the *V. cholerae* O139 *O*-specific antigen. (**A**) Retrosynthetic analysis of the *V. cholerae* O139 *O*-specific antigen (**B**) Linker installment at the monosaccharide stage to afford α-linked galactoside.

More recently, the Kovac group utilized a thiotolyl donor in conjunction with the α-directing effect of a 4,6-di-*tert*-butylsilylene group to stereoselectively install an azido linker en route to the *O*-specific antigen glycotope of *V. cholerae* O139 polysaccharide. A salient feature of this approach was the use of protecting groups amenable to global deprotection in the final step (Figure 13) [86]. Given the specific anomeric attachment of glycans found in Nature, synthetic strategies that produce either α- or β-anomeric isomers are essential. 

#### 2.2.1. *O*-Anomeric Functionalization with TMO Addition: Stepwise Introduction of Azide

**Figure 14:**
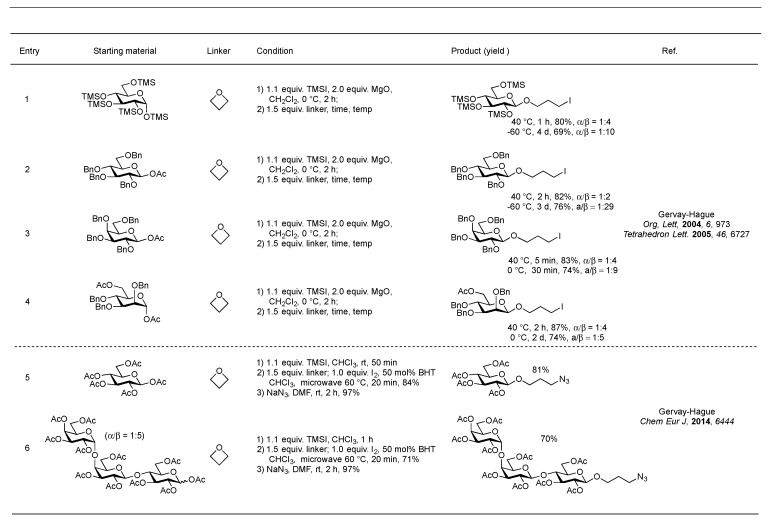
*O*-Anomeric functionalization using glycosyl iodide and trimethylene oxide (TMO).

The Gervay-Hague group has been interested in developing stereoselective glycosylation reactions using glycosyl iodides [30,87,88,89,90,91]. In early explorations, it was observed that glycosyl iodides generated in tetrahydrofuran readily underwent glycosylation yielding an iodo-terminated C-4 linker [92]. It was later discovered that strained cyclic ethers such as trimethylene oxide (TMO) readily add to armed glycosyl iodides to form β-selective 3-iodopropyl glycosides [93]. The methodology involves the formation of the glycosyl iodide using trimethylsilyl iodide (TMSI) in the presence of MgO, a weak base, which sequesters the formation of TMSOAc, followed by direct addition of TMO in various temperatures [94]. The α/β ratios of the glycosylated products ranged from 1:2 to 1:4 under reflux conditions and β-selectivity could be improved from 1:5 to 1:29 at lower reaction temperatures, albeit at the expense of longer reaction time. Nevertheless, the one-pot two-step glycosylation procedure afforded 69–89% yields under various conditions (Figure 14, entries 1–4). 

Extending the methodology to “armed” per-*O*-TMS or per-*O*-Bn oligosaccharides was not successful, as TMSI-promoted glycosyl iodide formation gave complex reaction mixtures due to internal glycosidic bond cleavage [30,95]. Efforts to employ per-*O*-acetylated sugars were more fruitful because ester protected analogs cleanly form stable glycosyl iodides. A recently developed iodide promoted two-step methodology for functionalizing per-*O*-acetylated sugars has been reported [96] (Figure 14, entries 5 and 6). The reaction involves in-situ iodide formation using TMSI and microwave I_2_-promoted TMO glycosylation to form the corresponding β-3-iodopropyl glycosides, which later on could be transformed to the corresponding azide using NaN_3_ at rt. The one-pot, step-economical and rapid functionalization was not only applied to per-*O*-acetylated monosaccharides but also di- and trisaccharides and no evidence of glycosidic bond cleavage was observed. The resulting iodopropyl glycosides were subsequently treated with sodium azide and subjected to global deprotection with NaOMe/MeOH to afford oligosaccharides with chemical handles for further modifications. With these azido handles in place, more sophisticated strategies can be applied to produce multivalent oligosaccharide containing antigens for therapeutic application.

#### 2.2.2. Multivalent Conjugation Strategies Using Azide-terminated Linkers

**Figure 15:**
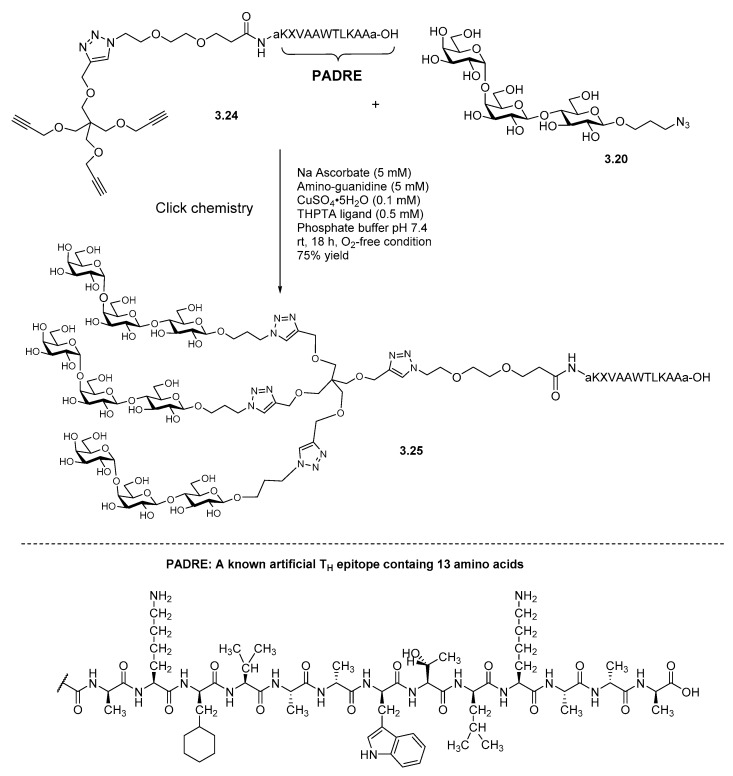
Multivalent vaccine candidate using azide terminated linkers.

The Gervay-Hague lab demonstrated using copper-catalyzed azide–alkyne cycloadditions to construct a new class of immune modulating molecules consisting of a trimeric carbohydrate moiety attached to a peptide epitope. PADRE, [97] a known artificial T helper (T_H_) epitope containing thirteen amino acids, has been used in cancer-vaccine development as an immunogenic carrier to stimulate the immune response. Applying the TMO-addition concept and click chemistry, a trimeric globotriaose–PADRE conjugate was constructed as a potential cancer-vaccine candidate (Figure 15). 

**Figure 16:**
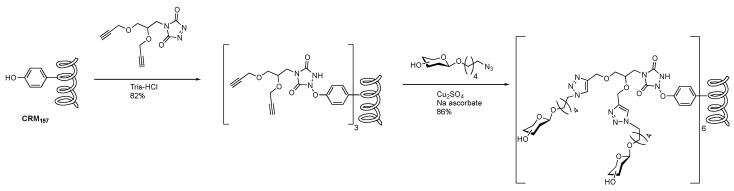
CuAAC mediated installment of sugars at predefined sites of CRM_197_ via site-selective tyrosine ligation [101].

More recently, researchers have exploited triazolinedione scaffolds to attach glycans to tyrosine residues of the genetically modified carrier protein, CRM_197_ [98,99,100,101], commonly present in FDA approved conjugate vaccines. Using the click approach, the Group B Streptococcus type II capsular polysaccharide was attached to a triazolinedione based linker, which was followed by a site-specific attachment to tyrosine moieties of CRM_197_ (Figure 16). Utilizing this technology, researchers at GSK Vaccines and Novartis Institutes for Biomedical Research (NIBR) analyzed efficacy vaccine candidates based upon the number of carbohydrates present and conjugation sites [101].

Since aberrant glycosylation is highly related to cancer, tumor-associated carbohydrate antigens (TACAs) become very promising synthetic targets for cancer vaccine development. To construct potential cancer vaccine candidates, several excellent reviews have been published to elaborate their design, synthesis and biology [38,39,102,103,104,105]. The above mention variants are all important to determine the potency of a vaccine candidate. The Danishefsky group was one of the pioneers in developing fully synthetic carbohydrate cancer vaccine. In 1999, the group synthesized 4-*n*-pentenyl fucosylated GM1 and successfully conjugated with KLH carrier protein. The construct had demonstrated high specificity to antibody against small cell lung carcinoma, suggesting its potential to elicit the immune response [48]. Along this line, the same group synthesized a series of functionalized TACAs, and then coupled them with immunogenic peptides as well as carrier proteins to construct multivalent TACA-containing cancer vaccines [64,106,107]. Multivalent glycol(cyclo)peptide are also a promising platform for cancer vaccine development [40]. The technology allows carbohydrates epitopes, immunogenic epitopes and proteins installed on the defined, size-controllable platforms, mimicking the multivalent nature on the cancer cell surface. 

Besides fighting cancer, multivalent carbohydrate vaccines have also been designed to treat bacterial and fungal infections [41,108,109]. Just like the approach with cancer vaccines, the constructs target particular oligosaccharides that are overexpressed on the bacterial or fungal surfaces, teaching the immune system to recognize the antigens. Alternative therapeutic strategies also include non-covalent multivalent constructs that utilize micelles and liposome formulations to trigger immune response [110]. The size and morphology of these aggregates can be controlled by introducing different lipid chains on the oligosaccharides, varying lipid compositions and adjusting physical conditions. The glycosylated liposomes are not only similar to the cellular environments (bio-compatible) but also have large surface area, which enhance the recognition interactions. 

## 3. Functionalized Sugars and Glycan Arrays

**Figure 17:**
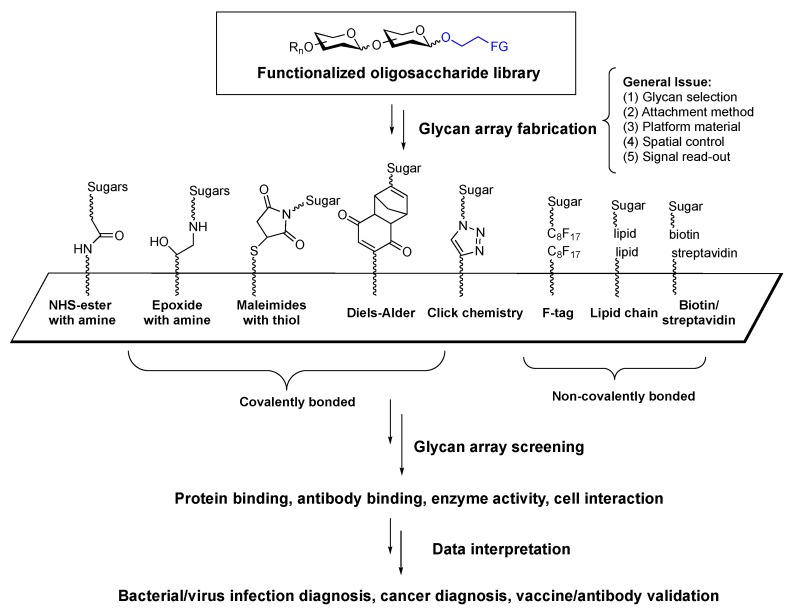
General scheme for sugar array preparation and application.

Functionalized sugars are great tools for chemical biology research and they are highly related to glycan array fabrication. It is known that the oligosaccharides in nature are highly diverse. They play an important role in cell-to-cell interaction, but detailed mechanisms of signaling processes are still unclear. Understanding the roles and the functions of the oligosaccharides has developed to a specialized area: Glycomics. To decipher the complex “sugar code”, understanding the binding interaction and the binding mode between sugars and other biomolecules is an obvious start. Since sugars are displayed outside of the cell surface, a platform for reconstructing the sugar-coated surface would be very useful to reflect the dynamics of the interaction. The idea of immobilizing functionalized oligosaccharide to form glycan arrays has been demonstrated by Wang [111], Mrksich [112], Feizi [113], and Wong [114] groups independently in 2002. Since then, the glycan arrays not only have become one of the major applications of functionalized oligosaccharides but also essential tools for studying glycobiology (Figure 17). 

Glycan arrays are powerful tools for the analysis of carbohydrate-related interactions, and their fabrications and applications are well reviewed in recent literature [42,115,116,117,118]. Since the screening required relatively small amounts of oligosaccharides and short equilibrium time, the technology enables scientists to extract useful kinetic data and analyze binding specificity among biomolecules such as proteins, antibodies and enzymes with reliable read-out. 

**Figure 18:**
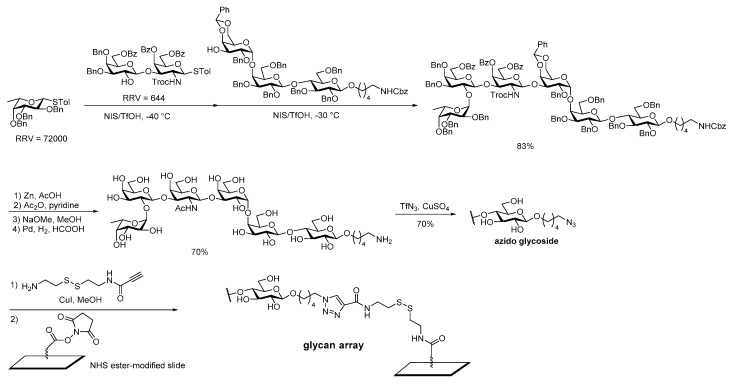
Wong’s total synthesis of azido-Globo H for glycan array.

In addition to carbohydrate-based vaccine development, glycan arrays have also benefitted from synthetic platforms that deliver carbohydrates equipped with functionalized linkers. An iterative one-pot chemical glycosylation to prepare complex oligosaccharides for immobilization on solid support was reported by Wong et al. [119]. Similar to Danishefsky’s 2nd generation globo H synthesis, they group chose to install a carboxybenzyl (Cbz) protected amine linker at the building block stage. The linker successfully went through the one-pot [1+2+3] glycosylation, as well as the global deprotection, affording globo H hexasaccharide with a terminal amine. Diazo transfer conditions developed in the Wong lab produced the azide that was then coupled with the disulfide alkyne linker via click chemistry. Subsequent reaction with an NHS-modified microplate successfully immobilized the globo H antigen on the solid support (Figure 18) [119].

## 4. Conclusions

Recent advances in the development of synthetic methodologies with wide scope and application have provided a more in-depth understanding of carbohydrate chemistry and glycobiology. While the atlas of human glycomics has yet been completed, having practical tools and materials, such glycolipid and glycopeptide conjugates, multivalent constructs and well defined glycan arrays, to tackle health related issues can bring meaningful insight to biomedical research and impact pharmaceutical science. 

A perennial issue in translational science involves obtaining sufficient amounts of well-characterized materials with high purity. While Nature produces highly complex and dense glycopeptides, these compounds are presented in scarce amounts making isolation and purification a significant challenge. Thus, synthetic methods and technologies to access these materials in large quantity and high quality are needed. Given that carbohydrates alone are generally poorly immunogenic, chemical strategies to conjugate peptides or carrier proteins in a site-specific manner are critical in carbohydrate-based pharmaceutical development. One effective approach incorporates a functionalized linker at the non-reducing ends of glycans as a functional handle for attaching peptides of interest. Selective methods for introducing the two most common linkers, terminal alkenyl and azido linkers, are described in this review. Taking advantage of functionalized oligosaccharides is a giant step toward discovering efficacious carbohydrate-based vaccines. Well-defined and characterized synthetic platforms will lead to better understanding of molecular recognition processes associated with diseases. 

Despite the aforementioned, several challenges still lie ahead. First, the structural diversity of currently available carbohydrates does not cover all of natural occurring oligosaccharides. To fully explore vaccine and glycan array development, new approaches to generate more structurally and connectively diverse functionalized oligosaccharides is necessary. Secondly, methods utilized in different laboratories are often difficult to replicate in laboratories with less expertise and the experimental read-outs may differ significantly. In order to have comparable analytical data, reliable standard operating procedures and processes need to be shared. For example, standardized glycan arrays would accelerate commercialization and mass production of this technology, bringing more impact to the scientific community. Analyzing complex and large high-throughput datasets generated by glycan array screening is not an easy task. The ability of extracting the meaningful data becomes crucial for interpretation experiment results. Therefore, developing methods to process the information is also warranted.
